# 4-Methyl-2,7-dioxo-3,6-dioxa-1(1,1′)-ferrocenacyclo­hepta­phane

**DOI:** 10.1107/S1600536811019398

**Published:** 2011-06-18

**Authors:** Xin Leng, Bingqin Yang, Pengfei Zhang, Xianwen Fang

**Affiliations:** a Key Laboratory of Synthetic and Natural Chemistry of the Ministry of Education, College of Chemistry and Materials Science, Northwest University of Xi’an, Taibai Bei Avenue 229, Xi’an 710069, Shaanxi, People’s Republic of China

## Abstract

In the title compound, [Fe(C_15_H_14_O_4_)], the two cyclo­penta­dienyl (Cp) rings are nearly parallel, making a dihedral angle of 2.6 (1)°. The distance between the centroids of the Cp rings is 3.309 (8) Å. The relative orientation of the two Cp rings is characterized by a torsion angle of −43.99 (6)° defined by the two centroids and the two substituted C atoms.

## Related literature

For the definition of ferrocenophanes, see: Otón *et al.* (2005 ▶). For the properties of ferrocenophanes, see: Cayuela *et al.* (2004 ▶); Kulbaba & Manners (2001 ▶); Lu *et al.* (2006 ▶); Mizuta *et al.* (2003 ▶); Nguyen *et al.* (1999 ▶); Otón *et al.* (2006*a* ▶,*b* ▶); Suzaki *et al.* (2006 ▶). For the synthesis and related structures, see: Gao *et al.* (2009 ▶); Leng *et al.* (2010 ▶). For studies of host structures for the investigation of mol­ecular recognition, see: Bond *et al.* (2009 ▶); Choi *et al.* (2006 ▶); Nakagaki *et al.* (2010 ▶). For a description of the Cambridge Structural Database, see: Allen (2002 ▶).
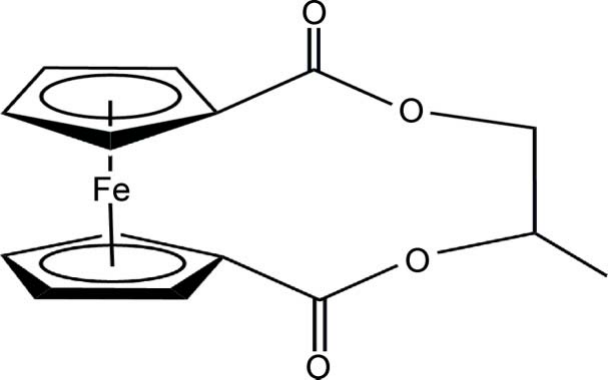


## Experimental

### 

#### Crystal data

[Fe(C_15_H_14_O_4_)]
*M* *_r_* = 314.11
Monoclinic, 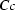

*a* = 7.1665 (14) Å
*b* = 20.131 (4) Å
*c* = 9.2464 (19) Å
β = 103.193 (2)°
*V* = 1298.7 (4) Å^3^
*Z* = 4
Mo *K*α radiation
μ = 1.17 mm^−1^
*T* = 296 K
0.29 × 0.21 × 0.12 mm

#### Data collection

Bruker APEXII CCD diffractometer
Absorption correction: multi-scan (*SADABS*; Sheldrick, 1996 ▶) *T*_min_ = 0.727, *T*_max_ = 0.870
3177 measured reflections
1971 independent reflections
1721 reflections with *I* > 2σ(*I*)
*R*_int_ = 0.024

#### Refinement

*R*[*F* ^2^ > 2σ(*F* ^2^)] = 0.036
*wR*(*F* ^2^) = 0.087
*S* = 0.96
1971 reflections
182 parameters
2 restraints
H-atom parameters constrained
Δρ_max_ = 0.30 e Å^−3^
Δρ_min_ = −0.41 e Å^−3^
Absolute structure: Flack (1983 ▶), 812 Friedel pairs
Flack parameter: 0.03 (3)

### 

Data collection: *APEX2* (Bruker, 2007 ▶); cell refinement: *SAINT* (Bruker, 2007 ▶); data reduction: *SAINT*; program(s) used to solve structure: *SHELXS97* (Sheldrick, 2008 ▶); program(s) used to refine structure: *SHELXL97* (Sheldrick, 2008 ▶); molecular graphics: *DIAMOND* (Brandenburg, 1999 ▶); software used to prepare material for publication: *SHELXL97*.

## Supplementary Material

Crystal structure: contains datablock(s) I, global. DOI: 10.1107/S1600536811019398/hy2432sup1.cif

Structure factors: contains datablock(s) I. DOI: 10.1107/S1600536811019398/hy2432Isup2.hkl

Additional supplementary materials:  crystallographic information; 3D view; checkCIF report

## Figures and Tables

**Fig. 1.**
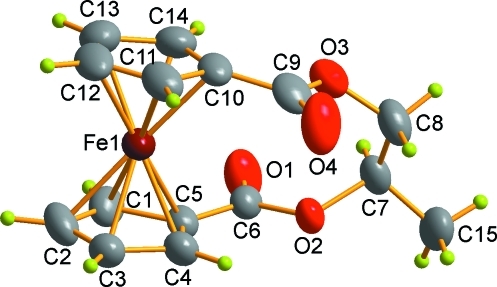
Molecular structure of the title compound. Displacement ellipsoids are drawn at the 50% probability level.

## supplementary crystallographic information

### Comment

Ferrocenophanes, in which the two cyclopentadienyl (Cp) rings are joined by an atomic or molecular bridge (Otón *et al.*, 2005), are found to be aromatic, highly stable and generally non-toxic, and have rversible redox characteristics (Mizuta *et al.*, 2003). In particular, ferrocenophanes are useful precursors to poly-ferrocenyl materials (Kulbaba & Manners, 2001; Nguyen *et al.*, 1999) and act as potentical receptor towards cation or anion recognition (Cayuela *et al.*, 2004; Lu *et al.*, 2006; Otón *et al.*, 2006*a*,b; Suzaki *et al.*, 2006). As a part of our ongoing investigation of ferrocenphanes, the title compound has been prepared and we report its crystal structure. Despite of the fact that structurally characterized ferrocenophanes are well presented in the Cambridge Structural Database (Allen, 2002; Version 5.27, release February 2009), there are only a few of structurally characterized compounds (Gao *et al.*, 2009; Leng *et al.*, 2010). Meanwhile, the study of the host structures is very helpful for the investigation of molecular recognition (Bond *et al.*, 2009; Choi *et al.*, 2006; Nakagaki *et al.*, 2010). From this viewpoint, X-ray single-crystal study of the title compound presents a certain descriptive interest.

The structure of the title compound is shown in Fig. 1. The two cyclopentadienyl (Cp) rings are nearly parallel, making a dihedral angle of 2.6 (1)°. The distance between the centroids of the Cp rings is 3.309 (8) Å. The angle formed between the two centroids and Fe1 is 179.4 (6)°. The relative orientation of the two Cp rings is characterized by the C6—*Cg*1—*Cg*2—C9 torsion angle of -43.99 (6)° (*Cg*1 and *Cg*2 are the centroids of C1–C5 ring and C10–C14 ring, respectively). The Fe—C distances range from 2.027 (5) to 2.073 (5) Å. The exocyclic C5—C6 and C9—C10 bond lengths are 1.456 (7) and 1.461 (9) Å.

### Experimental

The title compound was synthesized according to the published procedure (Gao *et al.*, 2009). Melting point, IR and NMR spectra confirmed identity and purity of the prepared compound.

Yellow crystals of the title compoud suitable for X-ray diffraction analysis were obtained by slow concentration of a dichloromethane solution at room temperature.

### Refinement

H atoms were positioned geometrically and refined using a riding model, with C—H = 0.96–0.98 Å and *U*_iso_(H) = 1.2(1.5 for methyl)*U*_eq_(C).

### Crystal data

**Table:**

[Fe(C_15_H_14_O_4_)]	*Z* = 4
*M**_r_* = 314.11	*F*(000) = 648
Monoclinic, *C**c*	*D*_x_ = 1.606 Mg m^−^^3^
Hall symbol: C -2yc	Melting point: 405(6) K
*a* = 7.1665 (14) Å	Mo *K*α radiation, λ = 0.71073 Å
*b* = 20.131 (4) Å	µ = 1.17 mm^−^^1^
*c* = 9.2464 (19) Å	*T* = 296 K
β = 103.193 (2)°	Block, yellow
*V* = 1298.7 (4) Å^3^	0.29 × 0.21 × 0.12 mm

### Data collection

**Table:**

Bruker APEXII CCD diffractometer	1971 independent reflections
Radiation source: fine-focus sealed tube	1721 reflections with *I* > 2σ(*I*)
graphite	*R*_int_ = 0.024
φ and ω scans	θ_max_ = 25.1°, θ_min_ = 2.0°
Absorption correction: multi-scan (*SADABS*; Sheldrick, 1996)	*h* = −8→8
*T*_min_ = 0.727, *T*_max_ = 0.870	*k* = −24→21
3177 measured reflections	*l* = −8→11

### Refinement

**Table:**

Refinement on *F*^2^	Secondary atom site location: difference Fourier map
Least-squares matrix: full	Hydrogen site location: inferred from neighbouring sites
*R*[*F*^2^ > 2σ(*F*^2^)] = 0.036	H-atom parameters constrained
*wR*(*F*^2^) = 0.087	*w* = 1/[σ^2^(*F*_o_^2^) + (0.0495*P*)^2^] where *P* = (*F*_o_^2^ + 2*F*_c_^2^)/3
*S* = 0.96	(Δ/σ)_max_ < 0.001
1971 reflections	Δρ_max_ = 0.30 e Å^−^^3^
182 parameters	Δρ_min_ = −0.41 e Å^−^^3^
2 restraints	Absolute structure: Flack (1983), 812 Friedel pairs
Primary atom site location: structure-invariant direct methods	Flack parameter: 0.03 (3)

### Fractional atomic coordinates and isotropic or equivalent isotropic displacement parameters (Å^2^)

**Table:**

	*x*	*y*	*z*	*U*_iso_*/*U*_eq_	
Fe1	0.13087 (9)	0.36266 (2)	0.68667 (8)	0.04130 (18)	
O1	0.5456 (6)	0.47208 (16)	0.8549 (4)	0.0654 (10)	
O2	0.5650 (4)	0.37627 (13)	0.9814 (3)	0.0426 (7)	
O3	0.2545 (5)	0.40433 (17)	1.0838 (4)	0.0562 (8)	
O4	0.1738 (6)	0.2959 (2)	1.0643 (5)	0.0820 (12)	
C1	0.3338 (7)	0.3998 (3)	0.5860 (6)	0.0522 (13)	
H1	0.3431	0.4459	0.5541	0.063*	
C2	0.2339 (10)	0.3478 (4)	0.4983 (7)	0.0609 (17)	
H2	0.1593	0.3520	0.3957	0.073*	
C3	0.2568 (7)	0.2893 (3)	0.5836 (6)	0.0523 (12)	
H3	0.2011	0.2458	0.5510	0.063*	
C4	0.3700 (7)	0.3047 (2)	0.7267 (5)	0.0480 (11)	
H4	0.4074	0.2732	0.8091	0.058*	
C5	0.4210 (9)	0.3728 (3)	0.7309 (7)	0.0404 (13)	
C6	0.5160 (6)	0.4129 (2)	0.8576 (5)	0.0408 (10)	
C7	0.5946 (7)	0.4116 (2)	1.1239 (5)	0.0465 (11)	
H7	0.5878	0.4596	1.1062	0.056*	
C8	0.4317 (8)	0.3902 (3)	1.1916 (7)	0.0585 (15)	
H8A	0.4410	0.3431	1.2142	0.070*	
H8B	0.4356	0.4144	1.2829	0.070*	
C9	0.1550 (7)	0.3512 (3)	1.0141 (6)	0.0547 (13)	
C10	0.0267 (9)	0.3693 (3)	0.8729 (7)	0.0475 (15)	
C11	−0.0857 (7)	0.3239 (3)	0.7722 (6)	0.0592 (14)	
H11	−0.1055	0.2768	0.7911	0.071*	
C12	−0.1631 (11)	0.3581 (4)	0.6428 (9)	0.069 (2)	
H12	−0.2445	0.3383	0.5536	0.083*	
C13	−0.1028 (7)	0.4244 (3)	0.6556 (7)	0.0655 (15)	
H13	−0.1372	0.4589	0.5793	0.079*	
C14	0.0168 (8)	0.4324 (3)	0.7992 (6)	0.0582 (13)	
H14	0.0788	0.4737	0.8414	0.070*	
C15	0.7879 (9)	0.3938 (3)	1.2184 (7)	0.0636 (16)	
H15A	0.7961	0.3465	1.2321	0.095*	
H15B	0.8050	0.4152	1.3133	0.095*	
H15C	0.8861	0.4083	1.1703	0.095*	

### Atomic displacement parameters (Å^2^)

**Table:**

	*U*^11^	*U*^22^	*U*^33^	*U*^12^	*U*^13^	*U*^23^
Fe1	0.0369 (3)	0.0492 (3)	0.0394 (3)	0.0023 (3)	0.0120 (2)	0.0010 (4)
O1	0.093 (3)	0.053 (2)	0.048 (2)	−0.0180 (17)	0.012 (2)	0.0077 (16)
O2	0.0533 (18)	0.0433 (16)	0.0294 (16)	0.0043 (13)	0.0056 (14)	−0.0023 (13)
O3	0.0564 (19)	0.066 (2)	0.049 (2)	−0.0042 (16)	0.0175 (17)	−0.0113 (16)
O4	0.095 (3)	0.086 (3)	0.059 (3)	−0.036 (2)	0.004 (2)	0.014 (2)
C1	0.046 (3)	0.071 (4)	0.043 (3)	−0.004 (3)	0.016 (2)	0.012 (3)
C2	0.058 (4)	0.090 (4)	0.038 (4)	−0.010 (3)	0.016 (3)	−0.011 (3)
C3	0.050 (3)	0.060 (3)	0.046 (3)	0.008 (2)	0.010 (2)	−0.008 (2)
C4	0.052 (3)	0.051 (3)	0.042 (3)	0.005 (2)	0.012 (2)	−0.006 (2)
C5	0.037 (3)	0.052 (3)	0.034 (3)	−0.002 (2)	0.011 (3)	−0.002 (2)
C6	0.039 (2)	0.049 (3)	0.036 (3)	0.0002 (19)	0.012 (2)	0.004 (2)
C7	0.062 (3)	0.044 (2)	0.032 (2)	−0.001 (2)	0.008 (2)	−0.0051 (18)
C8	0.067 (4)	0.072 (3)	0.036 (3)	−0.016 (3)	0.013 (3)	−0.007 (2)
C9	0.052 (3)	0.071 (4)	0.050 (3)	−0.015 (2)	0.028 (2)	−0.002 (2)
C10	0.031 (3)	0.074 (4)	0.041 (4)	0.000 (2)	0.014 (3)	−0.004 (2)
C11	0.045 (3)	0.079 (4)	0.057 (4)	−0.014 (3)	0.019 (3)	−0.016 (3)
C12	0.031 (3)	0.121 (7)	0.055 (5)	0.003 (3)	0.010 (3)	−0.015 (4)
C13	0.051 (3)	0.084 (4)	0.062 (4)	0.019 (3)	0.013 (3)	−0.002 (3)
C14	0.050 (3)	0.068 (3)	0.060 (4)	0.014 (2)	0.018 (3)	−0.009 (3)
C15	0.065 (3)	0.071 (4)	0.046 (3)	0.004 (3)	−0.005 (3)	−0.013 (3)

### Geometric parameters (Å, °)

**Table:**

Fe1—C14	2.027 (5)	C3—H3	0.9800
Fe1—C5	2.036 (6)	C4—C5	1.418 (7)
Fe1—C4	2.037 (5)	C4—H4	0.9800
Fe1—C10	2.032 (6)	C5—C6	1.456 (7)
Fe1—C1	2.040 (5)	C7—C15	1.503 (7)
Fe1—C12	2.054 (8)	C7—C8	1.508 (7)
Fe1—C13	2.053 (5)	C7—H7	0.9800
Fe1—C11	2.051 (5)	C8—H8A	0.9700
Fe1—C2	2.063 (6)	C8—H8B	0.9700
Fe1—C3	2.073 (5)	C9—C10	1.461 (9)
O1—C6	1.212 (5)	C10—C11	1.418 (7)
O2—C6	1.339 (5)	C10—C14	1.437 (7)
O2—C7	1.469 (5)	C11—C12	1.382 (10)
O3—C9	1.362 (6)	C11—H11	0.9800
O3—C8	1.452 (7)	C12—C13	1.401 (8)
O4—C9	1.202 (6)	C12—H12	0.9800
C1—C2	1.415 (9)	C13—C14	1.416 (8)
C1—C5	1.449 (8)	C13—H13	0.9800
C1—H1	0.9800	C14—H14	0.9800
C2—C3	1.406 (9)	C15—H15A	0.9600
C2—H2	0.9800	C15—H15B	0.9600
C3—C4	1.419 (6)	C15—H15C	0.9600
			
C14—Fe1—C5	109.9 (2)	C3—C4—Fe1	71.2 (3)
C14—Fe1—C4	136.5 (2)	C5—C4—Fe1	69.6 (3)
C5—Fe1—C4	40.76 (18)	C3—C4—H4	125.5
C14—Fe1—C10	41.5 (2)	C5—C4—H4	125.5
C5—Fe1—C10	112.3 (2)	Fe1—C4—H4	125.5
C4—Fe1—C10	109.9 (2)	C4—C5—C6	128.9 (5)
C14—Fe1—C1	113.2 (3)	C4—C5—C1	106.4 (5)
C5—Fe1—C1	41.7 (2)	C6—C5—C1	124.1 (4)
C4—Fe1—C1	68.6 (2)	C4—C5—Fe1	69.7 (3)
C10—Fe1—C1	143.3 (2)	C6—C5—Fe1	119.1 (4)
C14—Fe1—C12	67.5 (3)	C1—C5—Fe1	69.3 (3)
C5—Fe1—C12	176.8 (2)	O1—C6—O2	123.1 (4)
C4—Fe1—C12	142.4 (3)	O1—C6—C5	125.4 (4)
C10—Fe1—C12	67.1 (3)	O2—C6—C5	111.5 (4)
C1—Fe1—C12	137.1 (3)	O2—C7—C15	109.4 (4)
C14—Fe1—C13	40.6 (2)	O2—C7—C8	105.5 (4)
C5—Fe1—C13	136.9 (2)	C15—C7—C8	112.9 (5)
C4—Fe1—C13	176.9 (2)	O2—C7—H7	109.7
C10—Fe1—C13	68.5 (2)	C15—C7—H7	109.7
C1—Fe1—C13	111.0 (2)	C8—C7—H7	109.7
C12—Fe1—C13	39.9 (2)	O3—C8—C7	107.3 (5)
C14—Fe1—C11	68.7 (2)	O3—C8—H8A	110.2
C5—Fe1—C11	142.1 (2)	C7—C8—H8A	110.2
C4—Fe1—C11	113.0 (2)	O3—C8—H8B	110.2
C10—Fe1—C11	40.6 (2)	C7—C8—H8B	110.2
C1—Fe1—C11	175.7 (2)	H8A—C8—H8B	108.5
C12—Fe1—C11	39.3 (3)	O4—C9—O3	122.9 (5)
C13—Fe1—C11	67.6 (3)	O4—C9—C10	124.6 (5)
C14—Fe1—C2	142.9 (3)	O3—C9—C10	112.5 (5)
C5—Fe1—C2	68.8 (3)	C11—C10—C14	107.4 (5)
C4—Fe1—C2	67.8 (3)	C11—C10—C9	125.1 (5)
C10—Fe1—C2	175.4 (3)	C14—C10—C9	126.6 (5)
C1—Fe1—C2	40.3 (2)	C11—C10—Fe1	70.4 (3)
C12—Fe1—C2	112.0 (3)	C14—C10—Fe1	69.1 (3)
C13—Fe1—C2	114.0 (3)	C9—C10—Fe1	117.6 (4)
C11—Fe1—C2	135.9 (3)	C12—C11—C10	107.6 (6)
C14—Fe1—C3	176.6 (2)	C12—C11—Fe1	70.5 (4)
C5—Fe1—C3	68.4 (2)	C10—C11—Fe1	69.0 (3)
C4—Fe1—C3	40.38 (18)	C12—C11—H11	126.2
C10—Fe1—C3	136.0 (2)	C10—C11—H11	126.2
C1—Fe1—C3	67.7 (2)	Fe1—C11—H11	126.2
C12—Fe1—C3	114.3 (2)	C11—C12—C13	110.3 (7)
C13—Fe1—C3	142.6 (2)	C11—C12—Fe1	70.2 (4)
C11—Fe1—C3	110.7 (2)	C13—C12—Fe1	70.0 (4)
C2—Fe1—C3	39.7 (2)	C11—C12—H12	124.8
C6—O2—C7	117.2 (3)	C13—C12—H12	124.8
C9—O3—C8	116.9 (4)	Fe1—C12—H12	124.8
C2—C1—C5	107.9 (5)	C12—C13—C14	107.2 (6)
C2—C1—Fe1	70.7 (3)	C12—C13—Fe1	70.1 (4)
C5—C1—Fe1	69.0 (3)	C14—C13—Fe1	68.7 (3)
C2—C1—H1	126.0	C12—C13—H13	126.4
C5—C1—H1	126.0	C14—C13—H13	126.4
Fe1—C1—H1	126.0	Fe1—C13—H13	126.4
C3—C2—C1	108.6 (6)	C13—C14—C10	107.4 (5)
C3—C2—Fe1	70.5 (3)	C13—C14—Fe1	70.7 (3)
C1—C2—Fe1	68.9 (3)	C10—C14—Fe1	69.4 (3)
C3—C2—H2	125.7	C13—C14—H14	126.3
C1—C2—H2	125.7	C10—C14—H14	126.3
Fe1—C2—H2	125.7	Fe1—C14—H14	126.3
C2—C3—C4	108.0 (5)	C7—C15—H15A	109.5
C2—C3—Fe1	69.8 (3)	C7—C15—H15B	109.5
C4—C3—Fe1	68.4 (3)	H15A—C15—H15B	109.5
C2—C3—H3	126.0	C7—C15—H15C	109.5
C4—C3—H3	126.0	H15A—C15—H15C	109.5
Fe1—C3—H3	126.0	H15B—C15—H15C	109.5
C3—C4—C5	109.0 (5)		
			
C14—Fe1—C1—C2	−146.8 (4)	O2—C7—C8—O3	−55.2 (5)
C5—Fe1—C1—C2	118.9 (5)	C15—C7—C8—O3	−174.6 (4)
C4—Fe1—C1—C2	80.4 (4)	C8—O3—C9—O4	21.6 (7)
C10—Fe1—C1—C2	175.4 (5)	C8—O3—C9—C10	−157.7 (5)
C12—Fe1—C1—C2	−65.5 (6)	O4—C9—C10—C11	−3.3 (9)
C13—Fe1—C1—C2	−102.9 (4)	O3—C9—C10—C11	176.0 (5)
C3—Fe1—C1—C2	36.8 (4)	O4—C9—C10—C14	−171.4 (6)
C14—Fe1—C1—C5	94.3 (3)	O3—C9—C10—C14	7.9 (8)
C4—Fe1—C1—C5	−38.5 (3)	O4—C9—C10—Fe1	−87.9 (6)
C10—Fe1—C1—C5	56.4 (5)	O3—C9—C10—Fe1	91.5 (5)
C12—Fe1—C1—C5	175.6 (4)	C14—Fe1—C10—C11	118.4 (5)
C13—Fe1—C1—C5	138.2 (3)	C5—Fe1—C10—C11	−146.3 (3)
C2—Fe1—C1—C5	−118.9 (5)	C4—Fe1—C10—C11	−102.5 (3)
C3—Fe1—C1—C5	−82.1 (3)	C1—Fe1—C10—C11	176.9 (4)
C5—C1—C2—C3	−0.3 (7)	C12—Fe1—C10—C11	37.1 (4)
Fe1—C1—C2—C3	−59.5 (4)	C13—Fe1—C10—C11	80.3 (4)
C5—C1—C2—Fe1	59.2 (4)	C3—Fe1—C10—C11	−64.8 (5)
C14—Fe1—C2—C3	176.6 (4)	C5—Fe1—C10—C14	95.2 (4)
C5—Fe1—C2—C3	81.3 (4)	C4—Fe1—C10—C14	139.0 (3)
C4—Fe1—C2—C3	37.3 (3)	C1—Fe1—C10—C14	58.5 (5)
C1—Fe1—C2—C3	119.9 (5)	C12—Fe1—C10—C14	−81.4 (4)
C12—Fe1—C2—C3	−102.0 (4)	C13—Fe1—C10—C14	−38.1 (3)
C13—Fe1—C2—C3	−145.5 (3)	C11—Fe1—C10—C14	−118.4 (5)
C11—Fe1—C2—C3	−63.4 (5)	C3—Fe1—C10—C14	176.7 (3)
C14—Fe1—C2—C1	56.7 (6)	C14—Fe1—C10—C9	−121.4 (6)
C5—Fe1—C2—C1	−38.6 (3)	C5—Fe1—C10—C9	−26.2 (5)
C4—Fe1—C2—C1	−82.6 (4)	C4—Fe1—C10—C9	17.6 (4)
C12—Fe1—C2—C1	138.1 (4)	C1—Fe1—C10—C9	−62.9 (5)
C13—Fe1—C2—C1	94.6 (4)	C12—Fe1—C10—C9	157.2 (5)
C11—Fe1—C2—C1	176.7 (4)	C13—Fe1—C10—C9	−159.6 (5)
C3—Fe1—C2—C1	−119.9 (5)	C11—Fe1—C10—C9	120.1 (5)
C1—C2—C3—C4	0.7 (7)	C3—Fe1—C10—C9	55.3 (5)
Fe1—C2—C3—C4	−57.9 (4)	C14—C10—C11—C12	−0.7 (6)
C1—C2—C3—Fe1	58.6 (4)	C9—C10—C11—C12	−170.7 (6)
C5—Fe1—C3—C2	−82.4 (4)	Fe1—C10—C11—C12	−60.1 (4)
C4—Fe1—C3—C2	−120.0 (5)	C14—C10—C11—Fe1	59.4 (4)
C10—Fe1—C3—C2	177.4 (5)	C9—C10—C11—Fe1	−110.5 (6)
C1—Fe1—C3—C2	−37.3 (4)	C14—Fe1—C11—C12	80.1 (4)
C12—Fe1—C3—C2	95.8 (4)	C5—Fe1—C11—C12	175.4 (4)
C13—Fe1—C3—C2	58.5 (6)	C4—Fe1—C11—C12	−147.1 (4)
C11—Fe1—C3—C2	138.3 (4)	C10—Fe1—C11—C12	118.7 (5)
C5—Fe1—C3—C4	37.6 (3)	C13—Fe1—C11—C12	36.2 (4)
C10—Fe1—C3—C4	−62.6 (4)	C2—Fe1—C11—C12	−65.8 (6)
C1—Fe1—C3—C4	82.7 (3)	C3—Fe1—C11—C12	−103.5 (4)
C12—Fe1—C3—C4	−144.2 (4)	C14—Fe1—C11—C10	−38.7 (3)
C13—Fe1—C3—C4	178.5 (4)	C5—Fe1—C11—C10	56.7 (5)
C11—Fe1—C3—C4	−101.6 (3)	C4—Fe1—C11—C10	94.2 (3)
C2—Fe1—C3—C4	120.0 (5)	C12—Fe1—C11—C10	−118.7 (5)
C2—C3—C4—C5	−0.8 (6)	C13—Fe1—C11—C10	−82.5 (4)
Fe1—C3—C4—C5	−59.5 (4)	C2—Fe1—C11—C10	175.4 (5)
C2—C3—C4—Fe1	58.7 (4)	C3—Fe1—C11—C10	137.8 (3)
C14—Fe1—C4—C3	178.1 (4)	C10—C11—C12—C13	0.6 (7)
C5—Fe1—C4—C3	−119.6 (5)	Fe1—C11—C12—C13	−58.6 (5)
C10—Fe1—C4—C3	139.0 (3)	C10—C11—C12—Fe1	59.2 (4)
C1—Fe1—C4—C3	−80.3 (3)	C14—Fe1—C12—C11	−83.4 (4)
C12—Fe1—C4—C3	60.9 (5)	C4—Fe1—C12—C11	55.0 (6)
C11—Fe1—C4—C3	95.3 (3)	C10—Fe1—C12—C11	−38.3 (4)
C2—Fe1—C4—C3	−36.7 (3)	C1—Fe1—C12—C11	176.2 (4)
C14—Fe1—C4—C5	−62.3 (4)	C13—Fe1—C12—C11	−121.6 (6)
C10—Fe1—C4—C5	−101.4 (3)	C2—Fe1—C12—C11	136.8 (4)
C1—Fe1—C4—C5	39.3 (3)	C3—Fe1—C12—C11	93.5 (4)
C12—Fe1—C4—C5	−179.4 (6)	C14—Fe1—C12—C13	38.2 (4)
C11—Fe1—C4—C5	−145.1 (3)	C4—Fe1—C12—C13	176.7 (4)
C2—Fe1—C4—C5	82.9 (4)	C10—Fe1—C12—C13	83.3 (4)
C3—Fe1—C4—C5	119.6 (5)	C1—Fe1—C12—C13	−62.2 (6)
C3—C4—C5—C6	172.0 (5)	C11—Fe1—C12—C13	121.6 (6)
Fe1—C4—C5—C6	111.5 (6)	C2—Fe1—C12—C13	−101.6 (5)
C3—C4—C5—C1	0.6 (6)	C3—Fe1—C12—C13	−144.9 (4)
Fe1—C4—C5—C1	−59.9 (4)	C11—C12—C13—C14	−0.2 (8)
C3—C4—C5—Fe1	60.5 (3)	Fe1—C12—C13—C14	−58.9 (4)
C2—C1—C5—C4	−0.2 (6)	C11—C12—C13—Fe1	58.7 (5)
Fe1—C1—C5—C4	60.1 (4)	C14—Fe1—C13—C12	−118.6 (6)
C2—C1—C5—C6	−172.1 (6)	C5—Fe1—C13—C12	−179.7 (6)
Fe1—C1—C5—C6	−111.8 (6)	C10—Fe1—C13—C12	−79.7 (5)
C2—C1—C5—Fe1	−60.3 (4)	C1—Fe1—C13—C12	139.9 (4)
C14—Fe1—C5—C4	139.6 (3)	C11—Fe1—C13—C12	−35.7 (4)
C10—Fe1—C5—C4	95.1 (3)	C2—Fe1—C13—C12	96.1 (5)
C1—Fe1—C5—C4	−117.5 (5)	C3—Fe1—C13—C12	59.5 (6)
C13—Fe1—C5—C4	176.9 (4)	C5—Fe1—C13—C14	−61.1 (5)
C11—Fe1—C5—C4	59.0 (5)	C10—Fe1—C13—C14	38.9 (4)
C2—Fe1—C5—C4	−80.1 (4)	C1—Fe1—C13—C14	−101.5 (4)
C3—Fe1—C5—C4	−37.3 (3)	C12—Fe1—C13—C14	118.6 (6)
C14—Fe1—C5—C6	15.5 (5)	C11—Fe1—C13—C14	82.9 (4)
C4—Fe1—C5—C6	−124.1 (6)	C2—Fe1—C13—C14	−145.3 (4)
C10—Fe1—C5—C6	−29.0 (5)	C3—Fe1—C13—C14	178.1 (4)
C1—Fe1—C5—C6	118.4 (5)	C12—C13—C14—C10	−0.2 (7)
C13—Fe1—C5—C6	52.8 (6)	Fe1—C13—C14—C10	−60.0 (4)
C11—Fe1—C5—C6	−65.0 (5)	C12—C13—C14—Fe1	59.8 (4)
C2—Fe1—C5—C6	155.8 (5)	C11—C10—C14—C13	0.6 (6)
C3—Fe1—C5—C6	−161.4 (5)	C9—C10—C14—C13	170.3 (5)
C14—Fe1—C5—C1	−102.9 (4)	Fe1—C10—C14—C13	60.8 (4)
C4—Fe1—C5—C1	117.5 (5)	C11—C10—C14—Fe1	−60.2 (4)
C10—Fe1—C5—C1	−147.4 (3)	C9—C10—C14—Fe1	109.5 (6)
C13—Fe1—C5—C1	−65.6 (4)	C5—Fe1—C14—C13	140.5 (4)
C11—Fe1—C5—C1	176.6 (4)	C4—Fe1—C14—C13	178.4 (4)
C2—Fe1—C5—C1	37.4 (4)	C10—Fe1—C14—C13	−118.0 (5)
C3—Fe1—C5—C1	80.2 (3)	C1—Fe1—C14—C13	95.7 (4)
C7—O2—C6—O1	20.9 (6)	C12—Fe1—C14—C13	−37.6 (4)
C7—O2—C6—C5	−157.9 (4)	C11—Fe1—C14—C13	−80.1 (4)
C4—C5—C6—O1	−175.5 (5)	C2—Fe1—C14—C13	59.6 (6)
C1—C5—C6—O1	−5.5 (9)	C5—Fe1—C14—C10	−101.5 (4)
Fe1—C5—C6—O1	−89.0 (6)	C4—Fe1—C14—C10	−63.6 (5)
C4—C5—C6—O2	3.2 (8)	C1—Fe1—C14—C10	−146.3 (3)
C1—C5—C6—O2	173.3 (5)	C12—Fe1—C14—C10	80.4 (4)
Fe1—C5—C6—O2	89.7 (4)	C13—Fe1—C14—C10	118.0 (5)
C6—O2—C7—C15	−126.9 (5)	C11—Fe1—C14—C10	37.9 (3)
C6—O2—C7—C8	111.4 (5)	C2—Fe1—C14—C10	177.6 (5)
C9—O3—C8—C7	108.1 (5)		
